# Supplementary material for: The early predictive value of frailty for health-related quality of life among elderly patients with cancer receiving curative chemotherapy
Source: PLoS One. 2023 Aug 2;18(8):e0287320. doi: 10.1371/journal.pone.0287320 (PMC10395968; doi:10.1371/journal.pone.0287320)
Supplement: Attachment — Submitted filename: Response letter.docx [file pone.0287320.s002.docx]

**Responses to Reviewers’ Comments**

*PLOS ONE Education*

Manuscript ID: PONE-D-22-31278

Manuscript title: **Frailty as an Early Predictive Value for Health-Related Quality of Life among Elderly Cancer Patients Receiving Curative Chemotherapy**

We truly appreciate the opportunity to revise our manuscript titled, “**Frailty as an Early Predictive Value for Health-Related Quality of Life among Elderly Cancer Patients Receiving Curative Chemotherapy.**” The constructive and valuable comments from the reviewers have provided guidance, which we believe has improved the quality of our manuscript. The manuscript has been revised to address all the reviewers’ comments and concerns, including revisions for grammar and wording as well as the addition of references in response to the reviewers’ questions.

To facilitate the review, all changes to the manuscript are in **red** **bold type**. We firmly believe that the publication of this paper will make a significant contribution to the literature on the predictive value of frailty on the quality of life of elderly patients with advanced stages cancer. We hope that the manuscript is now acceptable for publication in *PLOS ONE*. Thank you very much for your time and attention.

**Response to Editor:**

| **Editor Comments** | **Response** |
| --- | --- |
| (1) Reviewer 1 pointed out the limitations of this study. Please revise the manuscript as suggested. | We have revised the manuscript according to the suggestions from reviewer 1. |
| (2) Health-Related Quality of Life could be affected by many factors, e.g., the condition of nutrition support (oral or tube enteral feeding), the need for stoma for stool passage, and the methods of chemotherapy (oral or intravenous route). The lack of complete data would lead to a bias in prediction results via this pre-chemotherapy frailty assessment. Please provide more information. | Thank you for such valuable advice. Health-related quality of life (HRQOL), as stated by the editor, is affected by many factors, such as nutritional support (oral or enteral feeding), the need for a stoma to defecate, and the method of chemotherapy (oral or intravenous). However, all patients in this study received traditional chemotherapy via injection. They can eat by mouth except for one patient with an NG tube. In addition, only three patients had a colostomy. Therefore, we believe the above factors have a limited effect on patients’ HRQOL. We have presented the above information in both the Results section **(P 11, L 205 – 207)** and the Discussion section **(P 21, L 316-329)**. |

**Response to Reviewer** **#1:**

| **Reviewer 1 Comments** | **Response** |
| --- | --- |
| This study aimed to investigate the early predictive value of pre-chemotherapy frailty assessment for post-chemotherapy health-related quality of life among elderly cancer patients receiving curative chemotherapy.  However, the study has several limitations that compromise its validity. The sample size is relatively small, while unknown cancer types are grouped together (we only know that 38% of the patients are reported to have lymphoma). There is no information on surgery and chemotherapy completeness and duration. The tested associations were not adjusted for important variables, like cancer type and stage (or it is not reported). Those factors can explain part of the variance in the post-chemotherapy quality of life, thus making present inferences not reliable. The methods and results are unclear and poorly reported. | Thank you for pointing out the purpose of our study.  Thank you for the constructive comments.  We listed cancer types and cancer stages in the Result section and in Table 2 **(P 11, L 197 – 202, and Table 2, P12)**. All patients received chemotherapy, and 109 of them with solid tumors received chemotherapy after surgery **(P 11, L 201-202)**.  In order to understand the unique effect of frailty on different domains of HRQOL, we controlled confounding variables first and then analyzed the effect of frailty on different domains of HRQOL **(P 10, L 179 - 186)**. |
| The authors should include a reference to the study from which the data was obtained as described in subsection 2.1 of the methods section. | Thank you for the suggestion.  Accordingly, the relevant reference has been added in subsection 2.1 **(P 7, L 132)**. |
| In section 2.4, the authors should clearly define what the "basic attributes" are and how they were selected for the final model. | Thank you for the suggestion.  Because experts suggest using scores from different domains of the HRQOL as the outcome variables for data analysis [1], the basic attributes selected for control were different on different domains of the HRQOL according to the results from univariate analysis. The sequence of variables that entered the hierarchical regression model was described in the statistical analysis section **(P 10, L 179- 186)**. The reviewer can find the basic attributes selected for control in step 1 in the Hierarchical regression model from Table 5 **(P 16 - 18)**. |
| In Table 2, the numbers for education do not add up to 178. The authors should explain if there were missing data and how they used this variable in the linear regression model. The same issue applies to the cancer stage variable. | Thank you for pointing out the mistake.  It is our mistake in Table 2. We have changed “45” to “61” for education **(P 12)**.  The missing data was very low in this study. However, if we find the missing data on outcome variables, we use the mean of that subscale to replace with the missing data. If the missing data happened in an independent variable, such as cancer stage (2 missing data), the regression model will choose those subjects who had the complete data on the independent and the dependent variables (N=176) for data analysis. |
| In section 3.2, rows 222-224, the meaning of the ">" sign in front of the frailty characteristics is not clear. The authors should avoid double reporting of the same numbers in the text and tables. | Thank you for the comment.  We have changed “>” to “,” **(P 13, L 216 - 217)**. The double-reported information in section 3.2 was deleted as well **(P 13, L 212 - 222)**. |
| In section 3.2, rows 227-230, it is unclear which frailty criteria were eventually used, five or seven domains? | Researchers suggested using 7 [2-6] or 5 [7-9] domains to perform the CGA. Since the prevalence of frailty was similar between seven domains and five domains of CGA assessment **(P 14, Table 4)**, we used 5 domains solution to indicate frailty for regression analysis **(P 15, L 242-253 & P20, L 292-298)**. |
| In section 3.3 and Table 5, the authors used paired t-tests to determine the significance of differences in HRQOL domains before and after chemotherapy. However, the distribution of the scores and their normality were not evaluated, which raises questions about the appropriateness of using paired t-tests. | Thank you for the reminder.  After careful consideration, we decide to delete Table 5 from the manuscript because it does not relate to our research question. |
| In section 3.3, the authors reported significant differences in 'mobility' and 'illness burden' QoL before and after chemotherapy, but not in 'future worries'. In section 3.4 and Table 6, linear regression was used to test the effects of demographic characteristics and frailty status on post-chemotherapy QoL for the domains of 'mobility', 'future worries', and 'illness burden'. The reasoning for this selection of domains is not clear. | Thank you for the reminder.  To reduce confusion in section 3.3, we removed information on comparing different domains of HRQOL as it was not relevant to our research aim.  There were 7 domains in HRQOL measuring by EORTC QLQ-ELD14. Originally, we listed 3 domains (mobility, future worries, and illness burden) which frailty was the significant predictor. After adding HRQOL at baseline as one of the confounding variables, frailty was found to be a significant predictor for the illness burden domain of HRQOL **(P15, L 242-253)**. After careful consideration, we decided to list all of the regression findings in Table 5 **(P 16 - 18)**. |
| In section 3.4, the concepts of "hierarchies" 1 and 2 should be clearly explained. Do they correspond to steps 1 and 2 from Table 6? It is also unclear if the associations were adjusted for baseline QoL. Furthermore, the selection of variables for the linear regression model is unclear. As it is reported in table 2, the authors collected more information about patients, than they included into models. Not clear why. | Thank you for the constructive comment.  After controlling for the basic variables with a significant effect on HRQOL and their HRQOL at baseline in step 1, the researcher included the presence or absence of frailty (using 5 domains of CGA) in step 2 to perform the regression analysis on various domains of HRQOL) **(P 15, L 242 – 253 & Table 5, P 16 - 18)**.  Although we collected more basic information in this study, only those basic attributes which had significant relationship with different domains of HRQOL were controlled in the regression model. |
| In Table 6, the steps 1 and 2 are not clearly specified. The authors should explain this clearly in the legend. The association is not adjusted for age, cancer type, and stage, which could have explained a part of the variance. | We explained clearly about the regression in both the statistical analysis section **(P10, L179-186)** and the result section **(P15, L 242-253 & Table 5, P16-18)**. In our study, age was not controlled in any domains of HRQOL because it did not have a significant relationship with any specific domain of HRQOL. In addition, cancer type was controlled for the family support domain of HRQOL, and cancer stage was controlled for the maintaining purpose domain of HRQOL **(Table 5, P16-18)**. |
| The discussion should focus on the findings of this study in relation to its aim, which is "the predictive value of pre-chemotherapy frailty assessment for post-chemotherapy health-related quality of life", rather than on comparing the descriptive characteristics of the cohort with other studies. | Thank you for the valuable comment.  In the latest review of the effect of frailty on the quality of life in elderly cancer patients, most scholars assess frailty after chemotherapy [10-13] to predict the quality of life and found that frailty is an important predictor of quality of life [10-13]. Additionally, most scholars use EORTC QLQ-C30 and FACT-G to measure the quality of life. However, the quality of life measurement tool (EORTC QLQ-ELD14) used in this study is rarely used by scholars, although it was developed by the European Organization for Research and Treatment of Cancer (EORTC) to evaluate the quality of life of elderly cancer patients. In addition, this study is the first one to predict the quality of life after chemotherapy (EORTC QLQ-ELD14) using frailty before chemotherapy in elderly cancer patients. Therefore, it is difficult to compare and discuss our results with other studies. It is possible that some of the variables which were not measured in this study, such as financial toxicity, treatment side effects, and spiritual well-being, may be related to quality of life, especially for elderly cancer patients in advanced stages **(P 21, L 316-329).**  We tried to explain the findings of this study through our clinical experience with cancer patients, in the hopes of meeting your expectations. |
| The statement "There was no statistically significant difference in most of the domains (p > .05), except for the mobility, future worries, and illness burden domains (p < .05)" in rows 385-387 contradicts the results reported in the results section "However, there were no statistically significant differences in other domains of HRQOL (worries about others, future worries, maintaining purpose, joint stiffness, and family support) (p >.05) (Table 5)". 'Future worries' was mentioned in both, which is inconsistent. | To decrease the confusion, we deleted the original Table 5 and its relevant information in section 3.3. In this study, frailty was found to be a significant predictor for the illness burden domain of HRQOL but not a significant predictor for other domains of HRQOL **(P15, L 242-253 & Table 5, P16-18)**. |

**Response to Reviewer #2:**

| **Reviewer ’s Comment** | **Response** |
| --- | --- |
| Thank you for the opportunity to review your paper I think it is well structured and asks a unique question. Thus i think it is a useful addition to the literature  I would recommend acceptance without modification | Thank you for your support. We truly appreciate it. |

**References**

1. Wheelwright S, Darlington AS, Fitzsimmons D, Fayers P, Arraras JI, Bonnetain F, et al. International validation of the EORTC QLQ-ELD14 questionnaire for assessment of health-related quality of life elderly patients with cancer. Br J Cancer. 2013;109(4):852-8. Epub 20130718. doi: 10.1038/bjc.2013.407. PubMed PMID: 23868003; PubMed Central PMCID: PMCPMC3749575.
2. Baitar A, Van Fraeyenhove F, Vandebroek A, De Droogh E, Galdermans D, Mebis J, et al. Evaluation of the Groningen Frailty Indicator and the G8 questionnaire as screening tools for frailty in older patients with cancer. J Geriatr Oncol. 2013;4(1):32-8. Epub 20120824. doi: 10.1016/j.jgo.2012.08.001. PubMed PMID: 24071490.
3. Owusu C, Koroukian SM, Schluchter M, Bakaki P, Berger NA. Screening older cancer patients for a Comprehensive Geriatric Assessment: A comparison of three instruments. J Geriatr Oncol. 2011;2(2):121-9. doi: 10.1016/j.jgo.2010.12.002. PubMed PMID: 21927633; PubMed Central PMCID: PMCPMC3173499.
4. Puts MT, Monette J, Girre V, Pepe C, Monette M, Assouline S, et al. Are frailty markers useful for predicting treatment toxicity and mortality in older newly diagnosed cancer patients? Results from a prospective pilot study. Crit Rev Oncol Hematol. 2011;78(2):138-49. Epub 20100504. doi: 10.1016/j.critrevonc.2010.04.003. PubMed PMID: 20444620.
5. Retornaz F, Monette J, Batist G, Monette M, Sourial N, Small D, et al. Usefulness of frailty markers in the assessment of the health and functional status of older cancer patients referred for chemotherapy: a pilot study. J Gerontol A Biol Sci Med Sci. 2008;63(5):518-22. doi: 10.1093/gerona/63.5.518. PubMed PMID: 18511757.
6. Valéro S, Migeot V, Bouche G, Raban N, Roullet B, Dreyfus B, et al. Who needs a comprehensive geriatric assessment? A French Onco-Geriatric Screening tool (OGS). Journal of Geriatric Oncology. 2011;2:130-6.
7. Mangia M, Dieli M, Arnoldi E, Labianca R, Minetti B. Comprehensive geriatric assessment (CGA): Gong (oncologic geriatric group), experience. ANNALS OF ONCOLOGY. 2005;16:68-.
8. Mohile SG, Bylow K, Dale W, Dignam J, Martin K, Petrylak DP, et al. A pilot study of the vulnerable elders survey-13 compared with the comprehensive geriatric assessment for identifying disability in older patients with prostate cancer who receive androgen ablation. Cancer. 2007;109(4):802-10. doi: 10.1002/cncr.22495. PubMed PMID: 17219443.
9. Wedding U, Ködding D, Pientka L, Steinmetz HT, Schmitz S. Physicians' judgement and comprehensive geriatric assessment (CGA) select different patients as fit for chemotherapy. Crit Rev Oncol Hematol. 2007;64(1):1-9. Epub 20070705. doi: 10.1016/j.critrevonc.2007.05.001. PubMed PMID: 17613243.
10. Kirkhus L, Harneshaug M, Šaltytė Benth J, Grønberg BH, Rostoft S, Bergh S, et al. Modifiable factors affecting older patients' quality of life and physical function during cancer treatment. J Geriatr Oncol. 2019;10(6):904-12. Epub 20190821. doi: 10.1016/j.jgo.2019.08.001. PubMed PMID: 31444088.
11. Kirkhus L, Šaltytė Benth J, Grønberg BH, Hjermstad MJ, Rostoft S, Harneshaug M, et al. Frailty identified by geriatric assessment is associated with poor functioning, high symptom burden and increased risk of physical decline in older cancer patients: Prospective observational study. Palliat Med. 2019;33(3):312-22. Epub 20190204. doi: 10.1177/0269216319825972. PubMed PMID: 30712456; PubMed Central PMCID: PMCPMC6376598.
12. Phaibulvatanapong E, Srinonprasert V, Ithimakin S. Risk Factors for Chemotherapy-Related Toxicity and Adverse Events in Elderly Thai Cancer Patients: A Prospective Study. Oncology. 2018;94(3):149-60. Epub 20171207. doi: 10.1159/000485078. PubMed PMID: 29212082.
13. Quinten C, Kenis C, Hamaker M, Coolbrandt A, Brouwers B, Dal Lago L, et al. The added value of geriatric assessment in evaluating a patient's Health-Related Quality-of-Life: A study in ≥70-year-old early-stage invasive breast cancer patients. Eur J Cancer Care (Engl). 2020;29(5):e13278. Epub 20200618. doi: 10.1111/ecc.13278. PubMed PMID: 32557904.
