# Supplementary material for: The early predictive value of frailty for health-related quality of life among elderly patients with cancer receiving curative chemotherapy
Source: PLoS One. 2023 Aug 2;18(8):e0287320. doi: 10.1371/journal.pone.0287320 (PMC10395968; doi:10.1371/journal.pone.0287320)
Supplement: Attachment — Submitted filename: Response to Editor.docx [file pone.0287320.s003.docx]

**Responses to Reviewers’ Comments**

*PLOS ONE Education*

Manuscript ID: PONE-D-22-31278

Manuscript title: **The Early Predictive Value of Frailty for Health-related Quality of Life Among Elderly Patients with Cancer Receiving Curative Chemotherapy**

We truly appreciate the opportunity to revise our manuscript again, titled “**The Early Predictive Value of Frailty for Health-related Quality of Life Among Elderly Patients with Cancer Receiving Curative Chemotherapy.**” The constructive and valuable comments from the editor have provided guidance, which we believe has improved the quality of our manuscript. The manuscript has been revised to address the editor’s comments and concerns, including revisions for grammar and wording as well as checking the references in response to the editor’s questions.

To facilitate the review, all changes to the manuscript are in **red** **bold type**. We firmly believe that the publication of this paper will make a significant contribution to the literature on the predictive value of frailty on the quality of life of elderly patients with advanced stages cancer. We hope that the manuscript is now acceptable for publication in *PLOS ONE*. Thank you very much for your time and attention.

**Response to Editor:**

| **Editor Comments** | **Response** |
| --- | --- |
| **Journal Requirements:**  Please review your reference list to ensure that it is complete and correct. If you have cited papers that have been retracted, please include the rationale for doing so in the manuscript text, or remove these references and replace them with relevant current references. Any changes to the reference list should be mentioned in the rebuttal letter that accompanies your revised manuscript. If you need to cite a retracted article, indicate the article’s retracted status in the References list and also include a citation and full reference for the retraction notice. | Thank you for the reminded. We have checked all of our references and ensured that no paper was retracted. Furthermore, we have re-checked and revised the reference style to make the style consistency. |
| **Additional Editor Comments:**   1. The authors have revised the text and tables based on the suggestions. 2. The similarity rate is 21%, which needs revision. 3. Please provide proof of English editing by a native English or a professional institution. | Thank you for your recognition!  Thank you for the comment. We have use *Turnitin* software, which can be used on similarity checking, and found that the similarity rate is 8% after excluding small matches (< 1%) (**Attachment 1**).  We have asked professional institution (*Editage*) to make sure this manuscript is concise and understandable and attach the editing proof in both the second page of the **Cover letter** and **supporting information (Attachment 2)**. |
